# Impact of initial active engagement in self-monitoring with a telemonitoring device on glycemic control among patients with type 2 diabetes

**DOI:** 10.1038/s41598-017-03842-2

**Published:** 2017-06-20

**Authors:** Min-Kyung Lee, Kwang-Hyeon Lee, Seung-Hyun Yoo, Cheol-Young Park

**Affiliations:** 1 Division of Endocrinology and Metabolism, Department of Internal Medicine, Myongji hospital, Seonam University College of Medicine, Gyeonggi-do, Korea; 2 Samsung Electronics Co. Ltd., Service R&D Team, Mobile Communications Business, Gyeonggi-do, Korea; 3 National Health Insurance Service, Seoul, Korea; 4 Division of Endocrinology and Metabolism, Department of Internal Medicine, Kangbuk Samsung Hospital, Sungkyunkwan University School of Medicine, Seoul, Korea

## Abstract

This study aimed to investigate the effect of patient engagement in self-monitoring with a telemonitoring device on glycemic control among patients with type 2 diabetes. We conducted a subanalysis of the telemonitoring device study in Kaiser Permanente Northern California members. We divided the telemonitoring group into 53 frequent and 54 infrequent users based on self-monitoring of blood glucose (SMBG) frequency of the first 6 weeks. The frequency of SMBG transmitted from the telemonitoring device was examined over 24 weeks. Clinic and laboratory tests were collected at baseline, 6 weeks and 6 months. There was no significant difference in baseline HbA1c level between the two groups. After 6 months, change in HbA1c was −2.4 ± 1.6% among frequent users and −1.5 ± 1.5% among infrequent users (p = 0.003). The proportion of patients achieving target HbA1C level at 6 months was significantly higher among frequent users than among infrequent users. An increased frequency of SMBG was significantly correlated with a reduction in HbA1c at 6 months. In conclusion, initial active engagement in self-monitoring with a telemonitoring device could provide incremental improvement of glycemic control over 6 months.

## Introduction

Diabetes mellitus is a chronic illness requiring continuous medical care^1^, and cases of uncontrolled diabetes or complications from diabetes are especially costly^2^. Glucose, blood pressure, and lipid control are important for preventing complications and reducing the cost associated with diabetes. Successful care of diabetes requires patient self-management, including adherence to medication, self-monitoring of glucose and blood pressure, and healthy lifestyle modifications^3^. Diabetes self-management is especially important among people with uncontrolled diabetes or with diabetes complications^4^. However, diabetes self-management is difficult to maintain, and ongoing education and support through frequent contact with diabetes care managers are needed^5^.

Telemonitoring systems have been introduced as a tool for managing type 2 diabetes mellitus^6^. In several clinical trials, telemonitoring interventions delivered via cellular phones and the internet have been shown to improve diabetes outcomes^7, 8^ and reduce diabetes care costs in the management of this chronic disease^9, 10^. However, health outcomes with telemonitoring systems among patients with diabetes have varied widely^11^, and a telemonitoring system alone is unlikely to improve outcomes^12^. Identifying the effectiveness of telemonitoring devices remains a challenge due to variability among patients’ self-management abilities. Patient engagement in type 2 diabetes self-management could be an important mediator in the effectiveness of telemonitoring devices^13^.

The Kaiser Permanente Northern California (KPNC) Medical Center in Santa Rosa, California, USA, conducted a 6-month randomized clinical study to evaluate the effectiveness of the Samsung Health Diary (SHD, Seoul, Korea) telemonitoring device among Kaiser Permanente members with type 2 diabetes. This study reported that the telemonitoring device did not demonstrate a beneficial effect on glycemic control^14^. We found that the frequency of telemonitoring varied among the participants. The frequency of self-monitoring of blood glucose (SMBG) transmitted from the telemonitoring device was tracked to assess patient engagement. We conducted a subanalysis to determine whether a telemonitoring device would be beneficial for patients engaging in more frequent SMBG. The objective of the study was to investigate the effect of initial engagement in self-monitoring with a telemonitoring device on glycemic control among patients with type 2 diabetes.

## Results

### Characteristics of the study population

The telemonitoring group was divided into 54 infrequent users and 53 frequent users based on the average SMBG frequency for the first 6 weeks. Table 1 shows the baseline characteristics of the standard care and infrequent and frequent user groups. There were no significant differences among the three groups in age, sex, weight, or body mass index (BMI) or in the outcome measures of systolic blood pressure (SBP), low-density lipoprotein cholesterol (LDL-C), fructosamine, or HbA1c levels. The mean HbA1c level was 9.2 ± 1.4% (77 ± 15 mmol/mol) among frequent users and 9.4 ± 1.4% (79 ± 15 mmol/mol) among infrequent users, and there was no significant difference in HbA1c level between the two groups (p = 0.461). The self-efficacy score was 161.6 ± 27.6 among frequent users, 142.0 ± 30.0 among infrequent users, and 144.8 ± 34.3 in the standard care group. The frequent users had significantly higher self-efficacy scores than did infrequent users (p < 0.001).

**Table 1:** Baseline clinical characteristics among the groups.

	Standard care group (*n* = 91)	Telemonitoring group			p
		Infrequent users (*n* = 54)	Frequent users (*n* = 53)	p^*^	
Age, yr	56.4 ± 8.7	53.5 ± 9.6	55.8 ± 9.9	0.225	0.209
Sex, male, % (*n*)	60.4 (55)	58.2 (32)	67.3 (35)	0.550	0.594
Weight, kg	104.4 ± 20.0	104.2 ± 20.8	104.4 ± 15.9	0.949	0.996
BMI, kg/m²	35.5 ± 6	35.5 ± 6.5	34.1 ± 6.4	0.264	0.408
SBP, mmHg	127.3 ± 17.1	126.8 ± 15.4	129.0 ± 16.1	0.343	0.776
LDL-C, mg/dL	88.4 ± 31.6	91.7 ± 35.4	90.6 ± 37.5	0.876	0.840
Fructosamine, µmol/L	324 ± 73.2	306.5 ± 57.8	324.8 ± 76.8	0.166	0.283
HbA1c, %	9.2 ± 1.5	9.4 ± 1.4	9.2 ± 1.4	0.461	0.602
Self-efficacy scale	144.8 ± 34.3^†^	142.0 ± 30.0^†^	161.6 ± 27.6	<0.001^‡^	0.002^‡^

Data are expressed as the mean ± SD. ^*^p value between telemonitoring groups. ^†^No differences between the groups with the same superscript symbol in post-hoc analyses. ^‡^p < 0.05.

### Change from baseline to 6 weeks and 6 months in the telemonitoring group

**Figure 1:**
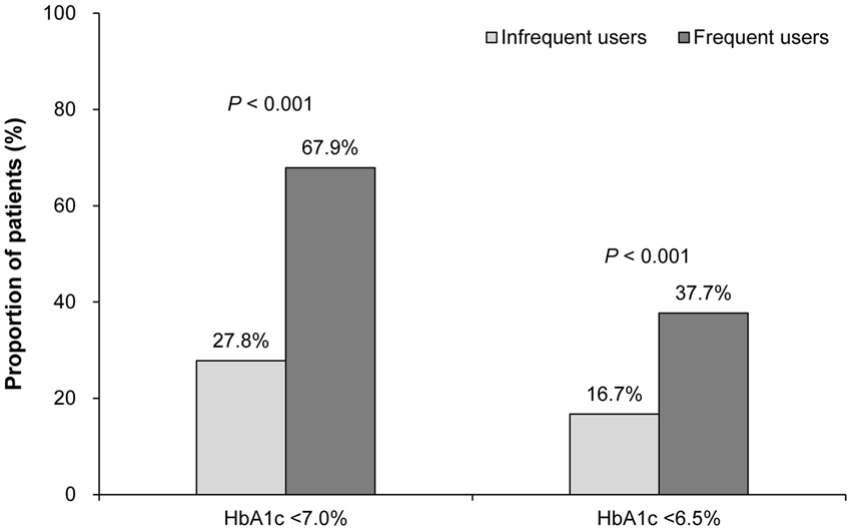
Proportion of patients achieving target HbA1C at 6 months.

Table 2 shows changes from baseline to 6 weeks and 6 months among the three groups. Frequent users showed the greatest improvement in SBP, LDL-C, and fructosamine levels at 6 weeks. At 6 months, HbA1C level improved significantly among frequent users relative to infrequent users and the standard care group, with mean changes in HbA1C of −2.4 ± 1.6% (−26 ± 17 mmol/mol), −1.5 ± 1.5% (−16 ± 16 mmol/mol) and −1.8 ± 1.7% (−20 ± 18 mmol/mol), respectively (p = 0.011). Fructosamine, which reflects glycemic control for the previous 2 to 4 weeks^15^, was significantly reduced among frequent users compared with infrequent users after 6 weeks (−70.1 ± 76.1 vs. 37.8 ± 44.2; p = 0.008); this gap increased after 6 months (−78.4 ± 81.2 vs. −20.5 ± 64.3; p < 0.001). In addition, at 6 months, there were no significant differences in the changes in BMI, SBP, LDL-C, and self-efficacy scores between frequent and infrequent users. We compared the attainment of the HbA1C target goal at 6 months in the two telemonitoring groups (Fig. 1). The proportion of patients achieving target HbA1C level at 6 months was significantly higher among frequent users than among infrequent users. The percentage of patients who achieved HbA1c < 7.0% at 6 months was 27.8% among infrequent users and 67.9% among frequent users (p < 0.001); 16.7% and 37.7% achieved HbA1c < 6.5%, respectively (p < 0.001).

**Table 2:** Changes from baseline to 6 weeks and 6 months among the groups.

	Standard care group (*n = *91)	Telemonitoring group			p
		Infrequent users (*n* = 54)	Frequent users (*n* = 53)	p^*^	
After 6 weeks from baseline					
Weight, kg	−2.4 ± 5.5	−1.7 ± 8.5	−3.3 ± 10.9	0.398	0.539
BMI, kg/m²	0.3 ± 4	0.2 ± 4.5	−0.1 ± 1.8	0.652	0.842
SBP, mmHg	−3.2 ± 4^†^	−0.7 ± 14.3^†^	−6.5 ± 13.9	0.035^‡^	0.021^‡^
LDL−C, mg/dL	−2.4 ± 20.4	−10.9 ± 76.1^†^	−15.2 ± 32.0^†^	0.704	0.012^‡^
Fructosamine, µmol/L	−59.4 ± 63^†^	−37.8 ± 44.2	−70.1 ± 76.1^†^	0.008^‡^	0.023^‡*^
After 6 months from baseline					
Weight, kg	−1.1 ± 11.4	0.3 ± 10.6	2.5 ± 18.2	0.445	0.516
BMI, kg/m²	−0.02 ± 1.2	0.0 ± 1.5	−0.1 ± 2.4	0.796	0.914
SBP, mmHg	−1.1 ± 16.8	−0.3 ± 16.1	−2.5 ± 17.9	0.505	0.789
LDL−C, mg/dL	−5.4 ± 28	−9.1 ± 32.8	−12.8 ± 35.5	0.576	0.387
Fructosamine, µmol/L	−51 ± 75	−20.5 ± 64.3	−78.4 ± 81.2	<0.001^‡^	<0.001^‡^
HbA1c, %	−1.8 ± 1.7^†^	−1.5 ± 1.5^†^	−2.4 ± 1.6	0.003^‡^	0.011^‡^
Self-efficacy scale	14.7 ± 33.1	10.6 ± 35.1	7.7 ± 22.1	0.611	0.417

Data are expressed as the mean ± SD. ^*^p value between the telemonitoring group. ^†^No differences between the groups with same superscript symbols in post-hoc analyses. ^‡^p < 0.05.

### Frequency of SMBG in the telemonitoring group

**Figure 2:**
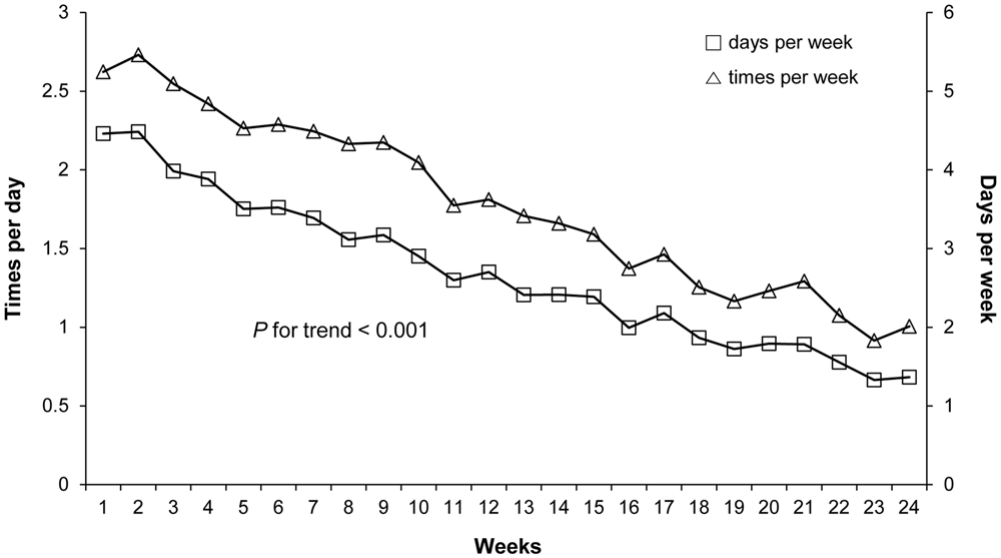
Frequency of weekly self-monitoring of blood glucose (SMBG) in the telemonitoring group. Data are expressed as mean ± SEM.

The telemonitoring participants performed SMBG an average of 1.99 ± 0.66 times per day for the first 6 weeks. Two participants (1.9%) never transmitted any SMBG data during the 24-week follow-up period. Another 25 participants (23.4%) performed SMBG on average < 1 time per day, and only one participant (0.1%) performed SMBG on average > 5 times per day; the remaining 79 participants (73.8%) performed SMBG between 1 and 4 times per day. The mean SMBG was testing 1.34 ± 0.98 times per day and 3.57 ± 2.03 days per week for 24 weeks. Figure 2 depicts the weekly SMBG frequencies ﻿in the telemonitoring group. SMBG frequency was 2.23 ± 1.43 times per day in the first week, 1.76 ± 1.35 at 6 weeks, and 0.68 ± 1.12 at 24 weeks. The fasting blood glucose level was 174.8 ± 50.95 mg/dl in the first week, 157.8 ± 49.13 at 6 weeks, and 142.2 ± 29.01 at 24 weeks. The weekly SMBG frequency gradually decreased over 24 weeks (p for trend < 0.001), and this was in concordance with the result as fasting blood glucose levels were relatively stable (p for trend < 0.001).

Table 3 shows the frequency of SMBG every 6 weeks for 24 weeks among frequent and infrequent users. The frequency of SMBG over the first 6 weeks was 2.88 ± 0.67 times per day among frequent users and 1.09 ± 0.65 among infrequent users. SMBG frequencies were significantly higher among frequent users than among infrequent users throughout the 24 weeks of follow-up.

**Table 3:** SMBG frequency between frequent users and infrequent users.

SMBG frequency	Times per day			Days per week		
	Infrequent users	Frequent users	p	Infrequent users	Frequent users	p
Total	0.77 ± 0.72	1.92 ± 0.86	<0.001	2.54 ± 1.95	4.7 ± 1.48	<0.001
Week 1 to 6	1.09 ± 0.65	2.88 ± 0.67	<0.001	3.53 ± 2.15	6.38 ± 0.76	<0.001
Week 6 to 12	0.89 ± 0.94	2.09 ± 1.03	<0.001	2.9 ± 2.51	5.25 ± 1.96	<0.001
Week 12 to 18	0.64 ± 0.94	1.57 ± 1.17	<0.001	2.04 ± 2.42	4.0 ± 2.21	<0.001
Week 18 to 24	0.45 ± 0.80	1.15 ± 1.14	<0.001	1.55 ± 2.28	2.91 ± 2.40	<0.001

Data are expressed as the mean ± SD.

### Association between SMBG frequency and glycemic control

In the telemonitoring group, the correlations between SMBG frequency and other variables (age, sex, weight, BMI, SBP, LDL-C, fructosamine, HbA1c, and self-efficacy score at baseline) were examined. Linear regression analysis revealed that age (β = 0.024; p = 0.020) and baseline self-efficacy scores (β = 0.010; p = 0.005) were significantly associated with SMBG frequency of the first 6 weeks (supplementary Table S1). In multiple linear regression analysis, SMBG frequency was significantly associated with reduction in HbA1c level at 6 months (Table 4). Changes in HbA1c at 6 months were highly associated with the frequency of SMBG for the first 6 weeks. In multivariable analyses, after adjusting for age, sex, and baseline self-efficacy score (model 2), SMBG frequency was also significantly associated with changes in HbA1c. In model 3, after adjusting for age, sex, BMI, SBP, LDL-C, HbA1c, and self-efficacy score at baseline, the results were the same as with model 2.

**Table 4:** Association between change in HbA1c at 6 months and SMBG frequency in the telemonitoring group.

	Crude		Model 1		Model 2		Model 3	
	β (95% CI)	p	β (95% CI)	p	β (95% CI)	p	β (95% CI)	p
SMBG frequency (days per week)								
Total	−0.444 (−0.760–−0.128)	0.006	−0.502 (−0.842–−0.163)	0.004	−0.522 (−0.862–−0.183)	0.003	−0.676 (−0.933–−0.420)	<0.001
Week 1 to 6	−0.458 (−0.744–−0.173)	0.002	−0.492 (−0.790–−0.195)	0.001	−0.626 (−0.841–−0.410)	<0.001	−0.444 (−0.889–−0.442)	<0.001
Week 6 to 24	−0.311 (−0.619–−0.002)	0.048	−0.364 (−0.704–−0.023)	0.037	−0.493 (−0.746–−0.239)	<0.001	−0.498 (−0.763–−0.233)	<0.001
SMBG frequency (days per week)								
Total	−0.263 (−0.410–−0.116)	0.001	−0.308 (−0.463–−0.153)	<0.001	−0.353 (−0.464–−0.241)	<0.001	−0.367 (−0.485–−0.249)	<0.001
Week 1 to 6	−0.263 (−0.397–−0.130)	<0.001	−0.295 (−0.433–−0.157)	<0.001	−0.330 (−0.427–−0.232)	<0.001	−0.352 (−0.455–−0.250)	<0.001
Week 6 to 24	−0.178 (−0.314–−0.041)	0.012	−0.211 (−0.358–−0.064)	0.005	−0.241 (−0.352–−0.130)	<0.001	−0.247 (−0.365–−0.129)	<0.001

Model 1 is adjusted for age and sex.

Model 2 is adjusted for age, sex, and baseline self-efficacy scale.

Model 3 is adjusted for age, sex, self-efficacy scale, BMI, SBP, LDL, and HbA1c at baseline.

## Discussion

This study evaluated the relationship between the frequency of SMBG based on data transmitted from a telemonitoring device and glycemic control. We found that an increased SMBG frequency for the first 6 weeks with the telemonitoring device was associated with improved glycemic control, as measured by HbA1c and fructosamine levels during the 6-month follow-up, among patients with type 2 diabetes. The telemonitoring device improved glycemic control early, late, and over the study period among frequent users who tested SMBG at least twice daily for the first 6 weeks. Initial active engagement in SMBG with a telemonitoring device was important in diabetes management.

This novel telemonitoring device was expected to provide an incremental benefit to diabetes management, but a previous study did not find that the telemonitoring device improved glycemic control above standard care^14^. The effects of telemonitoring systems can vary depending on patient engagement in diabetes self-management. In this study, we evaluated the effectiveness of a telemonitoring device when it was actively in use. We defined active users of the telemonitoring device based on the frequency of engaging in SMBG. A telemonitoring device could be a useful tool for improving the impact of self-monitoring by remotely linking patients with care managers^16^. It has also been reported that active care management provided via the telemonitoring device resulted in a significantly greater reduction in HbA1c level^17^. The telemonitoring device does not cure patients with diabetes, but active use of a telemonitoring device can be a significant factor in improvement of diabetes management and glycemic control.

In the current study, a majority (73.8%) of the telemonitoring group tested SMBG at least daily during the first 6 weeks, with an average of 1.99 times per day. This is much more frequent than what was observed in the National Health and Nutrition Examination Survey (NHANES)^18^. The frequency of SMBG gradually decreased in the telemonitoring group throughout the 24 weeks, but was consistently higher among frequent users than among infrequent users. SMBG is an integral component of diabetes therapy^1^, and structural SMBG is beneficial for patients with type 2 diabetes^19^; however, there is no guideline on the frequency of SMBG in non-insulin-treated type 2 diabetes^20^, and the added value of more frequent SMBG on glycemic control remains uncertain^21^. On the other hand, studies of insulin-treated type 1 diabetes have reported a correlation between SMBG frequency and glycemic control^22, 23^. Similarly, our study of patients with type 2 diabetes also showed that greater SMBG frequency was significantly correlated with lower HbA1c level at 6 months.

All of the participants in our research received appropriate interventions within the context of a close relationship with their healthcare managers. The increased contact between patients and healthcare providers through telemonitoring interventions can lead to improvements in diabetes management^24, 25^. Social support provided by healthcare workers can contribute to adherence among diabetic patients to such behaviors as following a diabetic diet, weight loss, taking prescribed medications, and checking blood glucose^26^. SMBG can be used to guide patients and healthcare providers in selecting appropriate pharmaceutical and lifestyle regimens to improve glycemic control on a day-to-day basis^27^. The previous study from the KPNC diabetes management also reported that patients initiating SMBG with a greater frequency had better glycemic control^28^. The current study indicated that SMBG frequency for the first 6 weeks could provide important feedback in order to improve long-term glycemic control among patients using the telemonitoring device.

We found that frequent SMBG during the initial engagement, not the whole study period, was associated with improved glycemic control. It will be difficult to sustain SMBG performance at least twice a day over a long period, due to patient compliance. We categorized frequent users as those who tested SMBG at least twice a day for the first 6 weeks, including measurement of fasting and postprandial glucose levels. However, our results cannot be a general guideline on the frequency of SMBG testing for type 2 diabetes patients. The current evidence suggests that SMBG frequency should be individualized depending on the clinical needs of individual patients and requirements of health providers^20, 29^. Considering this, it would be difficult to carry out a randomized controlled trial in which the study participants were allocated to different fixed SMBG frequencies for a certain period. A larger multicenter prospective cohort study might be a reasonable alternative approach for investigating the optimal SMBG frequency for patients with type 2 diabetes.

A recent study reported that a telemonitoring system might be helpful for improving patient engagement in SMBG and health outcomes^30^. The current study did not evaluate whether the telemonitoring device could positively affect patient engagement in SMBG due to the lack of SMBG data in the standard care group. We found that the frequency of SMBG was correlated with age and baseline self-efficacy score. The diabetes self-efficacy scale was developed based on self-care activities^31^. The concept of self-efficacy is relevant for improving self-management because diabetes self-management incorporates behavioral, personal, and environmental factors into daily performance of recommended activities. Several studies have reported that self-efficacy score was associated with diabetes self-management including SMBG^32–34^. However, the current study demonstrated that SMBG frequency was significantly associated with reduction in HbA1c even after adjusting for self-efficacy score. On the other hand, the frequency of SMBG was not related to cardiovascular risk factors including LDL, SBP, and BMI. Frequent users were more likely to achieve greater reduction in SBP at 6 weeks, but this difference was not significant at 6 months.

This study has several limitations. First, this is a post-hoc analysis, and the data from the telemonitoring group was collected from a relatively small sample. Nevertheless, the study was adequately powered for the outcomes examined and produced statistically significant results. Second, we adjusted for confounding variables through multivariable analysis. However, we could not completely rule out other factors related to patient engagement such as diabetic education, diet, physical activities, and medications. Finally, the study had a small sample size and short-term follow-up period, so it is uncertain whether these results can be sustained over 6 months with or without KPNC diabetes management. To solve these problems, a larger prospective long-term study is needed.

The present study showed that an initial active engagement in self-monitoring with a telemonitoring device could predict improved glycemic control throughout 6 months of follow-up among patients with type 2 diabetes. Patient engagement with a telemonitoring device could be a significant factor contributing to diabetes management. Therefore, initial strategies to enhance patient engagement in self-monitoring with a telemonitoring device are critical, and further research is necessary to determine the optimal use of a telemonitoring device.

## Methods

### Study population and design

The study targeted Kaiser Permanente members with type 2 diabetes who visited the Santa Rosa (SRO) Diabetes Care Management Center from May 2009 to April 2011. If patients agreed to participate, informed consent was obtained. A total of 198 members with poor glycemic control (HbA1c ≥ 7.5%) were randomly assigned to a standard care group (*n* = 91) or a telemonitoring group (*n* = 107). The telemonitoring group used the Samsung Health Diary (SHD, Seoul, Korea) telemonitoring device at home to transmit blood glucose, blood pressure, and weight measurements to a diabetes care manager. Diabetes care managers also used the telemonitoring device to deliver appropriate educational informational messages. In contrast, the standard care group received information from their care managers via e-mail, fax, and telephone. Participants in both groups received telephone consultations under the KPNC diabetes management program. Identification and recruitment of patients have been described in a previous study^14^.

We conducted a subanalysis of SMBG data from the telemonitoring group over 24 weeks; SMBG data from the standard care group was not available for the current study. We divided the telemonitoring group into infrequent users and frequent users based on average SMBG frequency for the first 6 weeks. Frequent users tested at least twice daily for the first 6 weeks. All participants visited the medical center for baseline randomization, followed by 6-week and 6-month follow-up clinic or laboratory tests and a self-administered self-efficacy questionnaire.

### The Kaiser Permanente Northern California (KPNC) diabetes program

KPNC Medical Center in Santa Rosa provided a traditional diabetes management program^14^. Both the standard care and telemonitoring groups received telephone consultations under this program. The 6-month care management program included an initial in-person office visit, followed by bi-weekly telephone calls. The major interventions were insulin self-titration, medication changes, and behavioral interventions. Medications were titrated for target goals based on data on patient glucose level sent to the medical center. Behavioral protocols included psycho/social referrals, diet, physical activity, smoking cessation, and others. Diabetes care managers provided appropriate interventions based on previously described target goals. Certified diabetes nurse educators, under the supervision of local endocrinologists and primary care physicians, were trained to use standard diabetes management protocols.

### Samsung Health Diary (SHD, Seoul, Korea) telemonitoring device

The Samsung Health Diary (SHD, Seoul, Korea) functions as a home-based gateway for obtaining measurement data, such as blood glucose, blood pressure, and weight, and records information to a server through an internet connection. The SHD transmits patient data to care managers; blood glucose and blood pressure are transmitted using a serial cable, whereas weight measurements are transmitted wirelessly. Diabetes care managers also use the telemonitoring device to deliver appropriate educational information to patients. In this study, the telemonitoring group received information automatically through the telemonitoring device as well as through web portal care.

### Outcomes

We evaluated the relationship between glycemic control and frequency of SMBG from the telemonitoring device to determine the utility of the SHD device among patients with type 2 diabetes. The primary outcome was to investigate whether increased SMBG for the first 6 weeks reduces HbA1C level at 6 months in both groups. The secondary outcome was to compare average fructosamine, weight, blood pressure, and LDL cholesterol measured at baseline, 6 weeks, and 6 months in all participants.

### Statistical analysis

All data were analyzed using IBM SPSS version 18.0 (IBM, Armonk, NY, USA) and were presented as mean ± standard deviation. We divided telemonitoring participants into frequent users and infrequent users according to frequency of SMBG and compared them to patients receiving standard care. The descriptive characteristics of the three groups were compared using either one-way analysis of variance (ANOVA) or t-tests for continuous variables, chi-square tests for categorical variables, and post hoc analyses with Tukey’s b method. The study outcomes were calculated as follow-up minus baseline values. Repeated-measures ANOVA was used to monitor how the frequency of SMBG changed over 24 weeks. Data is presented as mean ± standard error of mean. The frequency of SMBG in frequent users and infrequent users was compared using a t-test and was adjusted using the Bonferroni correction to reduce the chances of obtaining false-positive results (type I errors). Pearson’s correlation analysis was used to explore the correlations between SMBG frequency and other variables. Univariate and multivariate linear regression analyses were used to explore the association between change in HbA1c level and SMBG frequency.

This project was approved by the Kaiser Foundation Research Institute’s institutional review board. All methods were performed in accordance with the relevant guidelines and regulations.

Clinical trial registration number is NCT01775878, and the date of registration is January 23, 2013.

## Electronic supplementary material

Supplementary Table S1
