# Supplementary material for: Impact of initial active engagement in self-monitoring with a telemonitoring device on glycemic control among patients with type 2 diabetes
Source: Sci Rep. 2017 Jun 20;7:3866. doi: 10.1038/s41598-017-03842-2 (PMC5478652; doi:10.1038/s41598-017-03842-2)
Supplement: Supplementary file 1 — Supplementary Table S1 [file 41598_2017_3842_MOESM1_ESM.pdf]

Impact of initial active engagement in self-monitoring with a telemonitoring device on glycemic control among patients with type 2 diabetes

Min-Kyung Lee[1], Kwang-Hyeon Lee[2], Seung-Hyun Yoo[3], Cheol-Young Park [4*]

[1]Division of Endocrinology and Metabolism, Department of Internal Medicine, Myongji hospital, Seonam University College of Medicine, Gyeonggi-do, Korea

[2]Samsung Electronics Co. Ltd., Health R&D Group

[3]National Health Insurance Service, Seoul, Korea

[4]Division of Endocrinology and Metabolism, Department of Internal Medicine, Kangbuk Samsung Hospital, Sungkyunkwan University School of Medicine, Seoul, Korea

[*]**Corresponding Author:** Cheol-Young Park

Division of Endocrinology and Metabolism, Department of Internal Medicine, Kangbuk Samsung Hospital, Sungkyunkwan University School of Medicine, 29 Saemunan-ro, Jongno-gu, Seoul 03181, Korea

Tel: +82-2-2001-1869; Fax: +82-2-2001-1588

E-mail: cydoctor@chol.com

	β (95% CI)	p
Age, yr	0.024 (0.004-0.044)	0.020
Sex, male, % (n)	-0.164 (-0.576-0.249)	0.433
Weight, kg	0.001 (-0.004-0.005)	0.871
BMI, kg/m²	-0.010 (-0.004-0.005)	0.522
SBP, mmHg	0.005 (-0.008-0.018)	0.431
LDL-C, mg/dL	0.001 (0.197-0.844)	0.844
Fructosamine, µmol/L	0.001 (-0.002-0.004)	0.697
HbA1c, %	-0.097 (-0.214-0.020)	0.105
Self-efficacy scale	0.010 (0.003-0.017)	0.005

Supplementary Table S1. Linear regression analysis of factors associated with SMBG frequency of the first 6 weeks.
